# Rotating-disk electrode analysis of the oxidation behavior of dissolved Li_2_O_2_ in Li–O_2_ batteries†

**DOI:** 10.1039/c8ra03416h

**Published:** 2018-08-10

**Authors:** Jing Ren, Zhimei Huang, Pramod K. Kalambate, Yue Shen, Yunhui Huang

**Affiliations:** State Key Laboratory of Material Processing and Die & Mould Technology, School of Materials Science and Engineering, Huazhong University of Science and Technology (HUST) Wuhan 430074 Hubei P. R. China shenyue1213@hust.edu.cn huangyh@hust.edu.cn

## Abstract

The development of the rechargeable Li–O_2_ battery (LOB) has encountered several bottlenecks till date. One of the biggest challenges is to lower the oxidation potential of Li_2_O_2_, which is the insulating and insoluble discharge product. A possible solution to this problem is to use high acceptor number (AN) or donor number (DN) solvents to increase the solubility of Li_2_O_2_, so that the dissolved Li_2_O_2_ can diffuse to the cathode surface and get oxidized at a relatively low potential. Herein, we explored the efficiency and side-reactions in the LOB charge process with different Li_2_O_2_ soluble electrolytes. The relationship between the solubility of Li_2_O_2_ and charging rate was analyzed quantitatively with ultraviolet-visible (UV-Vis) spectroscopy and rotating disk electrode experiments. As a result, electrolytes with high AN usually have higher solubility for Li_2_O_2_ than electrolytes with high DN, and thus exhibit higher Li_2_O_2_ oxidation rates. Nevertheless, higher Li_2_O_2_ solubility in high AN electrolytes also induces more severe side reactions and easily passivates the electrode surface. The trade-off between charging reaction rate and electrolyte stability is a key issue to be considered when designing high performance LOB electrolytes.

## Abstract 2

The RDE method introduced in this study is a facile and informative technique to screen for high performance electrolytes for LOB.

## Graphical abstract

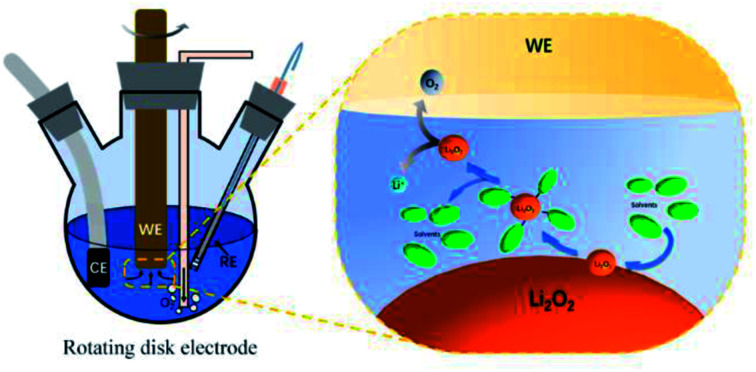

## 1 Introduction

**Fig. 1:**
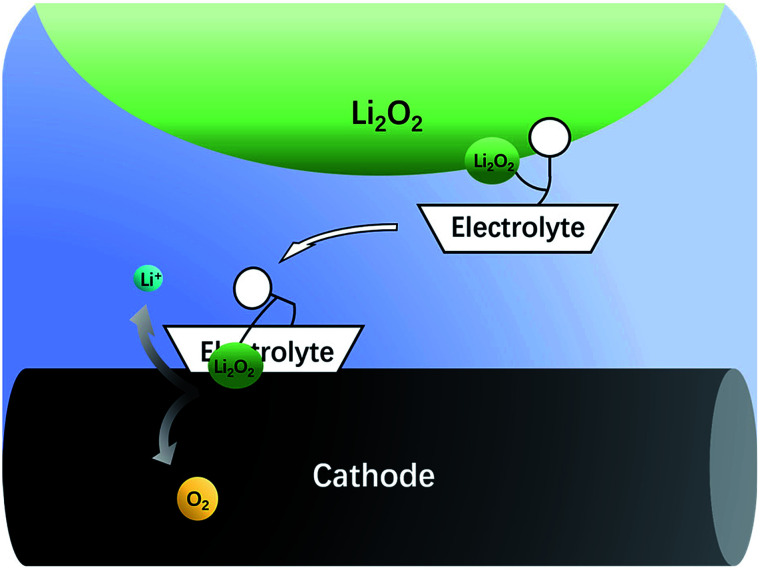
Electrolytes having ability to dissolve Li_2_O_2_ can transfer it to the surface of the cathode.

The nonaqueous LOB has been reported by Abraham *et al.* for more than 20 years.^1^ It has attracted much attention due to its high theoretic energy density.^2–8^ Its discharge and charge reactions are widely regarded to be 2Li + O_2_ ↔ Li_2_O_2_. Based on this mechanism, the theoretical energy density is as high as 3500 W h kg^−1^,^9,10^ which is the highest among all rechargeable batteries. However, the development of LOB suffers from numerous difficulties.^11–18^ One of the biggest problems is the insulating and insoluble nature of the Li_2_O_2_. During discharge, LiO_2_ is generated first *via* the oxygen reduction reaction. Then, the LiO_2_ is further reduced to get another Li^+^ to become Li_2_O_2_ at the surface of cathode,^19^ which is called a surface mechanism. Alternatively, it may dissolve in the electrolyte and generate the final product through disproportionation,^20,21^ which is called liquid nucleation. If the Li_2_O_2_ is not in close contact with the cathode, it is hard for Li_2_O_2_ to be oxidized back during the charge process because of the difficulty in the electron transfer process.^13,22,23^ As a result, the charge overpotential often exceeds 1 V and hence, Li_2_O_2_ cannot fully decompose. The high potential causes unwanted side reactions such as decomposition of the electrolyte, which is fatal to the battery.^8,16,24–27^ In the previous studies, solid catalysts supported on the cathode were first studied,^15,28–32^ but the poor solid–solid contact between Li_2_O_2_ and the cathode limited its catalytic efficiency. Then, liquid phase redox mediators were used to act as electron shuttles between Li_2_O_2_ and cathode,^33–42^ such as tetrathiafulvalene (TTF),^39^ LiI,^42^ 2,2,6,6-tetramethylpiperidinooxy (TEMPO),^34^ iron phthalocyanine (FePc),^40^ and tris[4-(diethylamino)phenyl]amine (TDPA).^41^ Recently, a promising novel solution to the abovementioned problem has been proposed, which uses electrolytes with high Li_2_O_2_ solubility to lower the charge potential.^43–46^ The principle is that Li_2_O_2_ is designed to dissolve in the electrolyte, so that it can diffuse to the cathode surface and be oxidized at a relatively low potential, as shown in Fig. 1. There are generally two types of Li_2_O_2_ soluble electrolytes, namely, the electrolytes with high acceptor number (AN) or high donor number (DN). In high AN electrolytes, such as electrolytes containing water, alcohol or phenol, the Li_2_O_2_ solubility is enhanced mainly due to strong solvation of O_2_^2−^.^46–48^ In high DN electrolytes, such as dimethyl sulfoxide (DMSO),^43^ dimethyl-2-imidazolidinone (DMI), hexamethyl phosphoryl triamide (HMPA),^44^ the solubility is also improved, mainly due to the solvation of Li^+^ ions.^49^ Both mechanisms are effective, but considering the complicated influence of the cathode architecture, catalyst and operation conditions, there is still a lack of quantitative study on the sole influence of the electrolyte.

In this study, the rotating disk electrode (RDE) was used for the first time to measure the oxidation efficiency of dissolved Li_2_O_2_ in different electrolytes. The advantage of the RDE is that it only reflects the effects of the electrolytes, and the influence of cathode, anode, and other components contained in the batteries can be effectively avoided. As a result, a huge difference in the oxidation current was observed between high AN and high DN electrolytes. High AN electrolytes generally have higher solubility of Li_2_O_2_, leading to higher currents. Nevertheless, significant cathode passivation was also observed, indicative of severe side-reactions on the cathode surface. The composition of these passivation films was extensively studied with X-ray photoelectron spectroscopy (XPS). According to these data, we propose that the formation rate of the passivation film strongly depends on the Li_2_O_2_ concentration. The dissolved Li_2_O_2_ would significantly increase the oxidation rate of the electrolyte. In order to further improve the reversibility of the LOB, a promising way is to develop a new electrolyte with high Li_2_O_2_ solubility and excellent anti-oxidation stability. Our research also indicates that RDE is a useful tool to screen the electrolytes used for LOB.

## 2 Experimental

### 2.1 Materials and reagents

Tetraethylene glycol dimethyl ether (TEGDME), butanol, dimethyl sulfoxide (DMSO), 1,3-dimethyl-2-imidazolidinone (DMI), and hexamethyl phosphoryl triamide (HMPA) were dried by molecular sieves (molecular sieves, 4 Å, Sigma Aldrich) for at least 1 week in a glove box. Bis(trifluoromethane) sulfonamide (LiTFSI) was used as the lithium salt (99.95%, Sigma Aldrich), which was thoroughly dried in a vacuum oven before dissolving in the electrolyte solvent. Phenol (99.5%, Sigma Aldrich) and Li_2_O_2_ (90%, Sigma Aldrich) were stored in a glove box. Lithium-ion conducting glass ceramic (LICGC, thickness ∼150 μm, OHARA Inc. Japan) was used to protect the reference electrode. Concentrated sulfuric acid (98%, Sinopharm Chemical Reagent Co., Ltd, China) and titanyl sulfate (Sigma Aldrich) were used for UV-Vis studies.

### 2.2 RDE experiment

The RDE experiment was performed on a Pine RDE system (Pine Research Instrumentation, U.S.A.). All tests were conducted on a CHI-660E electrochemical workstation (CH Instruments, Shanghai, China). A golden disk electrode with 5 mm diameter was used as the working electrode (WE). A piece of stainless steel sheet coated with lithium titanium phosphate (LTPO) was used as the counter electrode (CE). A lithium foil in 0.5 M LiTFSI/TEGDME protected by LICGC was used as the reference electrode (RE). Specific production methods of the RE are as follows. First, LICGC was cut into a plate of about 5 × 5 mm^2^ and placed on one side of quartz glass tube and slightly fixed *via* a UV curing adhesive. Second, the junction between LICGC and the tube was sealed and mechanically enhanced using epoxy adhesive (EC-1838, 3M Co. Ltd.). Third, a piece of lithium foil pressed on stainless steel mesh was inserted into the tube. Then, 0.5 M LiTFSI/TEGDME was injected into the tube and sealed (the amount of injected liquid should immerse the lithium foil). All the assembly processes were carried out in the glove box. The experimental facility is shown in Fig. S1.† Before testing, the working electrode was polished with 0.05 μm alumina slurry and rinsed with deionized water. Linear sweep voltammetry (LSV) was performed in O_2_ atmosphere to mimic a LOB's environment. Each ventilation time was about 30 minutes. Various 0.5 M LiTFSI/electrolytes mentioned above with and without saturated Li_2_O_2_ were tested between 2.8-3.8 V (*vs.* Li/Li^+^) at a scan rate of 10 mV s^−1^.

### 2.3 UV-Vis titration test

UV-visible titration experiments were used to quantitatively measure the solubility of lithium peroxide in different solvents. The experiments were performed on a UV-Vis spectrophotometer (V-650, Jasco). Before testing, 100 mL of 1 M H_2_SO_4_ containing 2 g Ti(iv)OSO_4_ was prepared as the original solution after stirring to transparency. Then, 22.6 mg Li_2_O_2_ was dissolved in 10 mL of the above original solution. [Ti(O_2_)]^2+^ was formed after Li_2_O_2_ was added to the solution, which showed different shades of color when the concentration of O_2_^2−^ changed. The generated [Ti(O_2_)]^2+^ showed the absorption peak at *λ* = 405 nm in the UV-Vis absorption mode. According to Lambert–Beer law, *A* = *Kbc* (where *A* is the absorbance, *K* is molar absorption coefficient, *b* is the absorption layer thickness, and *c* is concentration of the coloring substances), the absorption intensity is proportional to the concentration of Li_2_O_2_. We diluted 10, 20, 50, 100 and 150 μL dissolved solution to 5 mL as standard samples. Subsequently, these five standard samples were measured on the instrument. The solubility of Li_2_O_2_ in different solutions can be obtained accurately by using five standard solutions for references.

To study the effect of solvents, excess lithium peroxide was added to different solvents and stirred for 24 h. Then, these solvents were centrifuged at 1200 rad s^−1^ for 10 min and certain amounts of supernatants were taken out, mixed with original solution, and tested by UV-Vis.

### 2.4 Characterization equipments

The electrode after LSV study was investigated by XPS (ESCALAB 250Xi XPS system) with a monochromatic Al X-ray source (1486.6 eV). The acquired XPS spectra were calibrated with the adventitious carbon peak (1 s) as a reference positioned at 285 eV.

## 3 Results and discussion

### 3.1 Solubility of lithium peroxide in different solvents

The concentration of Li_2_O_2_ in different solvents was researched by UV-Vis, as mentioned in the experimental section. When different solvents saturated with Li_2_O_2_ was added to the Ti(iv)OSO_4_ solution, the color of the solution changed into different shades of yellow. Considering the possible influences of the solvents, pure solvents were first mixed with the original solution (Fig. S2a†). No significant color change was observed compared to the original solvents. These solutions were also investigated by UV-Vis spectroscopy (Fig. S2b†). No absorption peak was observed, except in case of DMI and HMPA, which showed slight absorption at around 400 nm. This is because the solvent itself has a slight yellowish color. This absorption is too small to be ascribed to the absorption of dissolved lithium peroxide, so it has almost no influence on the results.

**Fig. 2:**
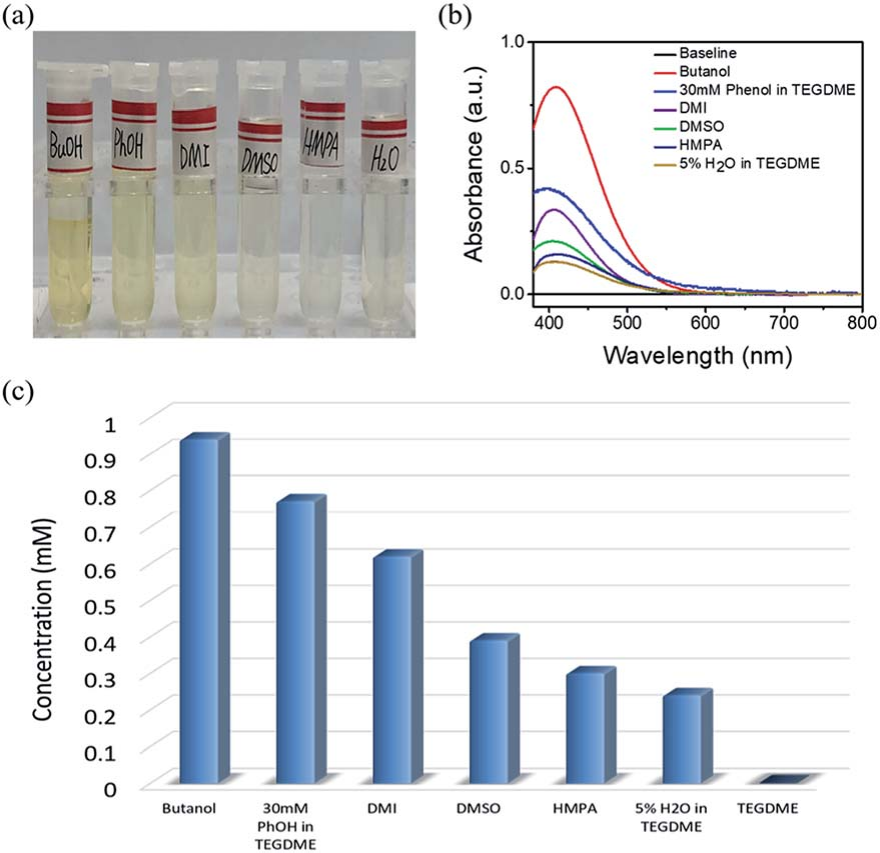
Digital photo (a) and UV-Vis absorption spectrum (b) of Li_2_O_2_ dissolved in different electrolytes. (c) The solubility of Li_2_O_2_ in different electrolytes.

The standard samples and solvents-dissolved Li_2_O_2_ were also investigated by UV-Vis spectroscopy, as shown in Fig. S2c and S2d†. According to the result of the standard samples, a straight line can be fitted for the relationship between concentration and absorbance. Therefore, the concentration of lithium peroxide in different solvents can be obtained by absorbance (Fig. 2a and b). It is clear that the solubility of Li_2_O_2_ in high AN solvents is higher than that in high DN solvents. The concentrations in butanol (9.36 × 10^−4^ mol L^−1^) and 30 mM phenol/TEGDME (7.74 × 10^−4^ mol L^−1^) are more than twice the concentrations in DMSO (3.94 × 10^−4^ mol L^−1^) and HMPA (2.95 × 10^−4^ mol L^−1^), indicating that high AN solvents are more capable of solvating Li_2_O_2_ than high DN solvents. The comparison of different solvents can be intuitively observed in Fig. 2c. To our knowledge, H_2_O is also a proton-donating substance, but its proton donating ability is weaker than that of phenol. Moreover, the concentration of H_2_O is limited, so the concentration of Li_2_O_2_ in 5% H_2_O/TEGDME is relatively small.

### 3.2 Oxidation efficiency of dissolved Li_2_O_2_ in different electrolytes

**Fig. 3:**
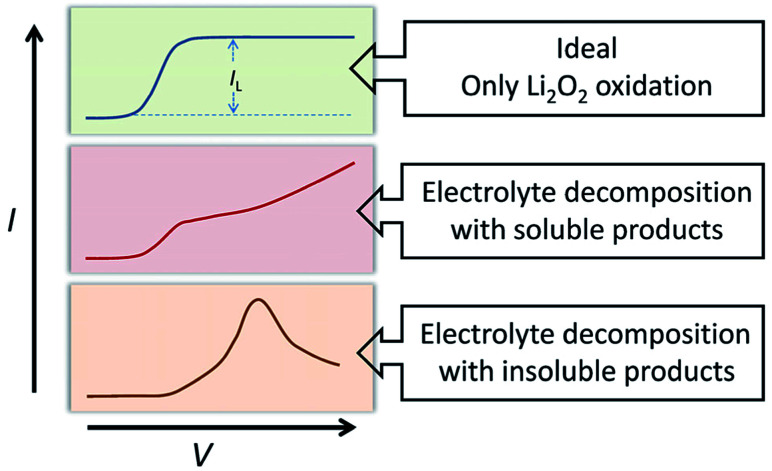
Schematic of comparison of ideal situation and actual situation in LSV test.

**Fig. 4:**
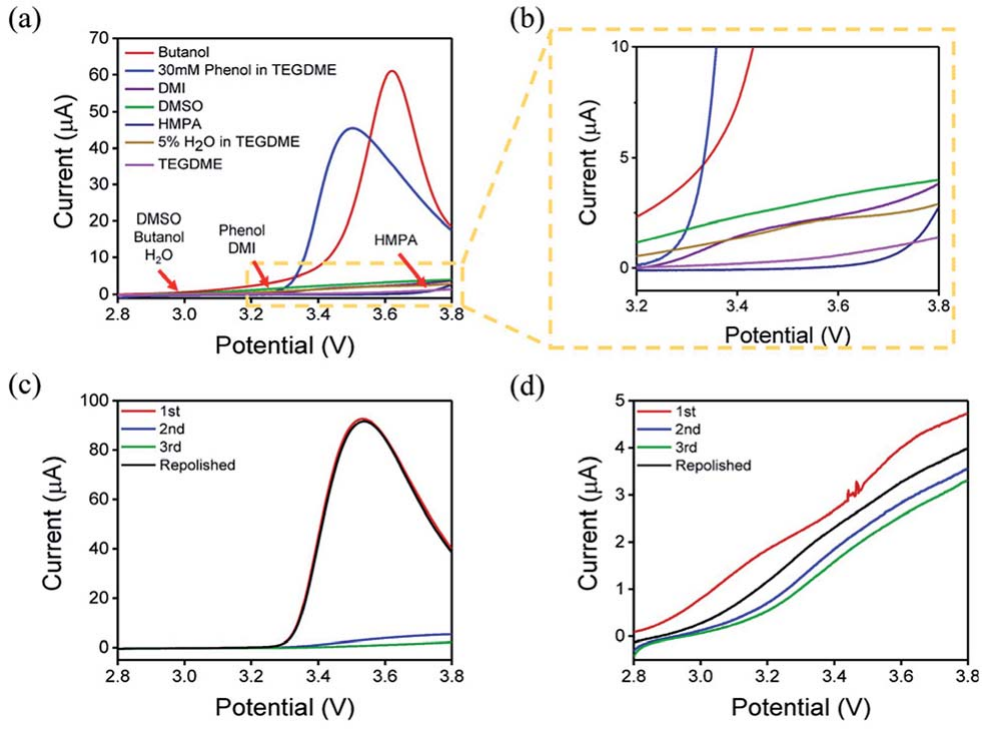
(a) LSV curves of different electrolytes at the potential range of 2.8–3.8 V (the onset potentials of different solvents are marked). (b) Enlarged view inside the dotted line. Successive sweep of AN electrolytes (c) and DN electrolytes (d). The current decreased with the increase in the scan times (3 times in front) and returns to the level as the first scan after the electrode was repolished (4^th^ curve).

The oxidation reaction of Li_2_O_2_ was investigated by RDE measurements. Ideally, the dissolved Li_2_O_2_ will continuously flow to the electrode with the rotation of the electrode. When bias voltage is applied on the electrode, the Li_2_O_2_ will begin to oxidize at a specified potential. As the electrode keeps rotating, Li_2_O_2_ constantly flows to the electrode surface, so the concentration of Li_2_O_2_ can remain saturated. If there are no other side reactions, the oxidation current will rise and finally maintain a constant value. In the actual situation, however, due to the disparity in oxidation efficiency of Li_2_O_2_ and the side reactions, there was a difference in oxidation current between the AN and DN electrolytes. If most of the by-products are soluble, they cannot passivate the electrode surface and hence, the rate of the side reaction is potential controlled. Thus, the current keeps increasing as the potential increases. Moreover, if most of the by-products are insoluble, they will passivate the electrode, causing the current to drop rapidly after rising to a maximum, as shown in Fig. 3. Hence, when the potential increases, the curve can reflect the ability of the electrolytes to dissolve lithium peroxide and the effect of the side reactions. All the selected solvents were measured by LSV from 2.8 to 3.8 V (*vs.* Li/Li^+^), which is a normal potential range for LOB. The pure electrolytes were measured first to see if they can be oxidized in this potential range, as shown in Fig. S3.† All the electrolytes are stable in this range. Then, the dissolution of Li_2_O_2_ in the electrolytes was studied. As shown in Fig. 4a, Li_2_O_2_ is oxidized as the potential increases. The oxidation current represents the oxidation efficiency of Li_2_O_2_. It is clear that oxidation current in high AN solvents is much larger than that in high DN solvents, which indicates that the ability of high AN solvents to solvate lithium peroxide is better than high DN solvents. High AN solvents should be weak acids according to the “Hard Soft Acid Base” theory. AN solvents have the ability to donate protons. Protons donated by solvents will chemically combine with peroxide ions from Li_2_O_2_ to form HOOLi, which then further transforms into large particles of Li_2_O_2_ in solution. The reaction process is as follows: Li_2_O_2(s)_ + AH → HOOLi_(sol)_ + A^−^ + Li^+^. High DN solvents have the ability to donate electrons, which can combine with Li^+^ from Li_2_O_2_ and its intermediates to form strong bonds. Li^+^ acts as a strong acid and the formed Li^+^-(solvent)_*n*_ can decrease the acidity of Li^+^; hence, the O^2−^ and O_2_^2−^ in solution are more easily stabilized by Li^+^-(solvent)_*n*_. AN solvents have stronger ability to dissolve Li_2_O_2_ than DN solvents because the combination of the AN solvents with Li_2_O_2_ tends to be a chemical combination, but the DN solvents combine with Li_2_O_2_ in a physical manner. Moreover, the large amount of Li^+^ present in the solvents will reduce the effect of solvating Li_2_O_2_ and its intermediates in high DN solvents. Therefore, the effect of high AN solvent-solvated lithium peroxide is better than that of DN solvent, but at the same time, there will be more O_2_^2−^ in the solution to attack the electrolyte and cause side reactions.

The parasitic reactions in the high AN solvents are more evident than those in high DN solvents, which can be verified in the results of the RDE study. In ideal conditions, the results obtained for the same solvent should be similar for every LSV study. However, we found in the experiment that with an increase in number of scans, the current value of each scan will reduce. As shown in Fig. 4c and d, the current of the solvents decreases with the increase in scanning times, and the current drop in the AN solvent is extreme, indicating that the oxidation reaction on the electrode surface was significantly reduced, probably due to the passivation of the electrode surface. Considering that the oxidation of Li_2_O_2_ does not passivate the electrode surface, the passivation is more likely caused by the side-product from the electrolyte oxidation. This phenomenon indicates that as the reaction progresses, by-products are generated and deposited on the electrode surface, which will passivate the electrode surface and reduce the current. In order to verify this situation, the electrode was polished and cleaned after several scans and reused for the study. Apparently, the current returned to the level as the first scan. This result proves that there is indeed a build-up of by-products on the electrode surface.

### 3.3 Analysis of side effects in different solvents

As mentioned in the previous section, parasitic reactions indeed occur in either AN or DN solvents, and are more intense in high AN solvents. In a highly reactive oxidizing environment filled with oxygen and its active state ions (O_2_^2−^, O^2−^), solvents are easily attacked. In high DN solvents, the solvents will combine with Li^+^ and reduce its acidity, which is beneficial for stabilizing O_2_^2−^ and O^2−^. However, high AN solvents can be easily attacked by the dissolved peroxide and superoxide species. In order to understand the effects of side reactions, the charged electrode was subjected to XPS analysis.

The process for the side reactions in butanol is proposed in the following reaction (1). In the strong oxidizing environment, carbon linked to hydroxide is vulnerable to be attacked by oxygen radicals, while CH_3_(CH_2_)_3_O^−^ will be partially oxidized and converted to C=O and eventually oxidized to Li_2_CO_3_.1
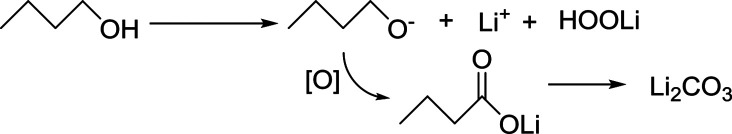


**Fig. 5:**
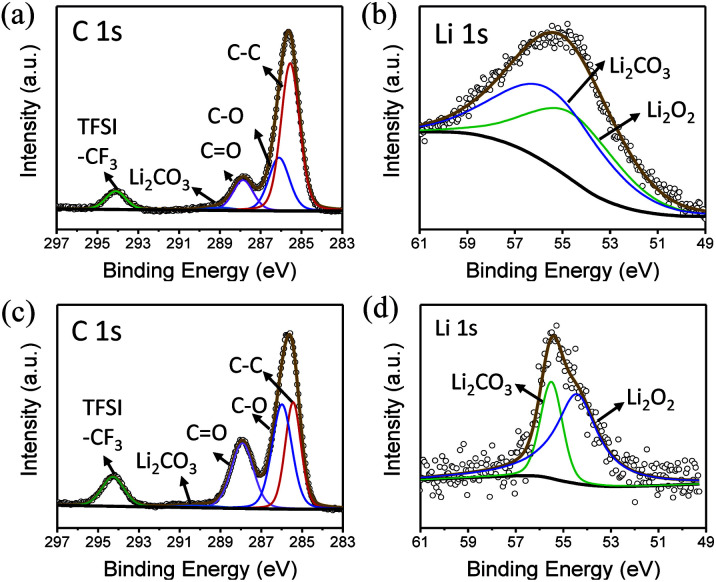
(a) C 1s and (b) Li 1s XPS spectra of the electrode surface in butanol. (c) C 1s and (d) Li 1s XPS spectra of the electrode surface in phenol/TEGDME.

To explore the composition of the by-products, XPS was performed (Fig. 5a and b). In the C 1s spectrum, peaks belonging to C=O and Li_2_CO_3_ can be clearly observed at 287.9 and 288.9 eV, respectively. Similarly, the presence of Li_2_CO_3_ can be detected in Li 1s spectra, indicating that butanol is decomposed. In addition, LiF is observed at 686 eV in F 1s spectrum, which shows that the lithium salt is not absolutely stable in this environment (Fig. S4†).

In the phenol/TEGDME solvent, no XPS peak for the aromatic ring was observed on the charged electrode, indicating that the passivation layer is not composed of the oxidation product of phenol. However, the side reactions brought about by TEGDME are evident, as shown in the reaction (2). When the ether chain is broken during the reaction, CH_3_OOLi is obtained. Then, CH_3_OOLi is further oxidized to form aldehyde and eventually, Li_2_CO_3_ is formed.2
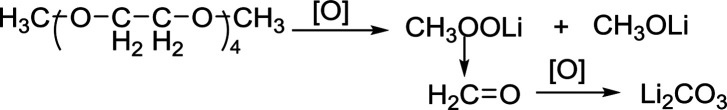


From the XPS C 1s spectrum (Fig. 5c), it can be seen that the peaks of C=O and Li_2_CO_3_ are clearly located at 287.9 eV and 288.9 eV, and the presence of Li_2_CO_3_ can also be detected in the Li 1s spectra (Fig. 5d). Moreover, a small amount of LiF can also be seen in the F 1s spectrum. Similarly, in 5% H_2_O/TEGDME, the parasitic reactions arise from oxidation of TEGDME.

The situation in the DN solvents is different. Since DN solvents can help stabilize the O_2_^2−^ and O^2−^, the side reactions of DN solvents are less than those in AN solvents. DMSO has been reported as a high DN solvent for a long time. The side reaction is displayed in the reaction (3); H in –CH_3_ can be captured by oxygen radicals, and then the C=S bonds are activated. Finally, DMSO_2_ is generated and LiOH is achieved at the same time.3
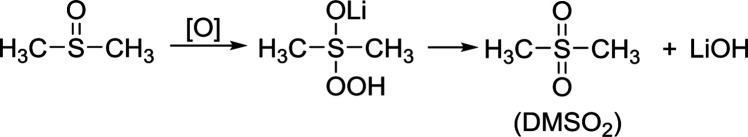


**Fig. 6:**
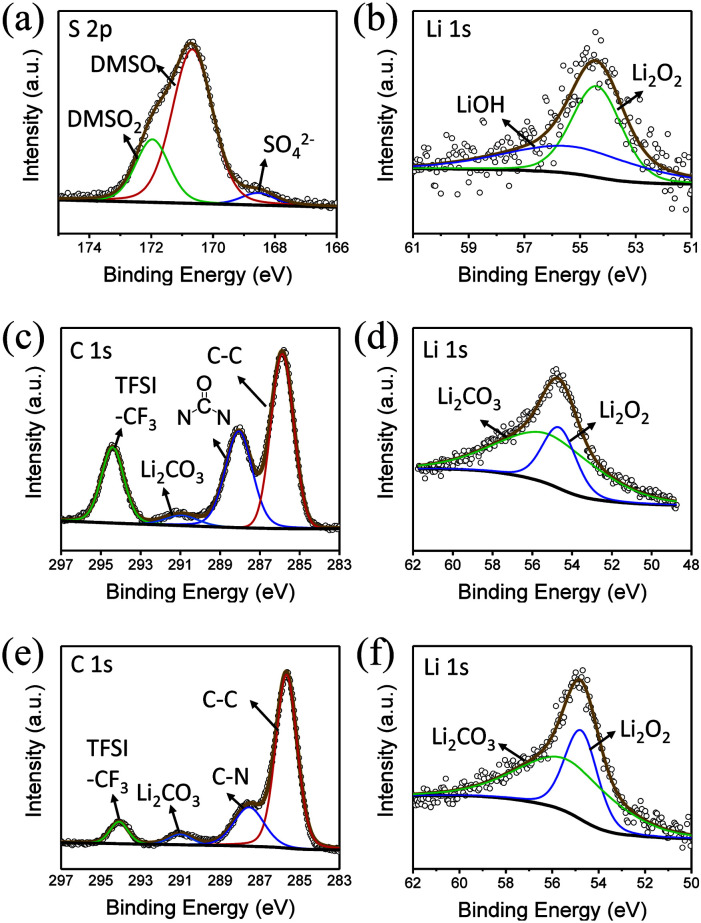
(a) S 2p and (b) Li 1s XPS spectra of the electrode surface in DMSO. (c) C 1s and (d) Li 1s XPS spectra of the electrode surface in DMI. (e) C 1s and (f) Li 1s XPS spectra of the electrode surface in HMPA.

In the S 2p spectrum (Fig. 6a), the peak at the position of 169.8 eV, corresponding to DMSO_2_, can be seen, demonstrating that DMSO is oxidized. At the same time, a small peak of SO_4_^2−^ appears at 167 eV, which is also the result of oxidation of DMSO. Generation of LiOH can also be detected in the Li 1s spectra (Fig. 6b).

DMI, as a high DN solvent, has not been previously reported, but similar urea solvents have been used in LOB before. The N–CH_2_ of DMI is the easiest to be attacked. Therefore, 1,3-dimethylurea and lithium oxalate will be obtained first; then, lithium oxalate will be further oxidized to obtain Li_2_CO_3_, as shown in the reaction (4). In C 1s and Li 1s XPS patterns (Fig. 6c and d), the peak of Li_2_CO_3_ is detected, which proves the formation of by-products.4
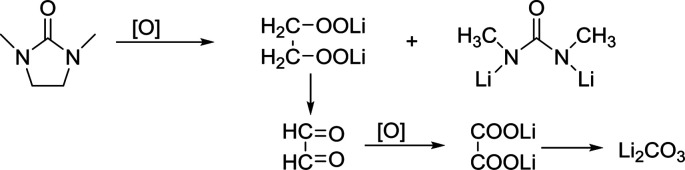


In comparison, HMPA is more stable in this environment, though its ability to solvate Li_2_O_2_ is not strong enough. The side reaction of HMPA is proposed in reaction (5). The central P atom is protected by the surrounding –O and –N(CH_3_)_2_ from the attack of O_2_^2−^ and O^2−^, but the N–C bond may be partly disassembled. As seen from the C 1s and Li 1s spectra (Fig. 6e and f), signs of C–N's separation can be observed and Li_2_CO_3_ is the final by-product.5
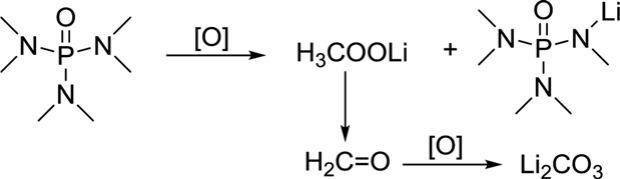


According to the abovementioned results, decomposition occurred in all the selected electrolytes, indicating that the stability of electrolyte and the solubility of Li_2_O_2_ are the key considerations for selecting electrolytes.

## 4 Conclusions

We have studied the oxidation behavior of dissolved Li_2_O_2_ in different electrolytes. High AN electrolytes generally have higher solubility of Li_2_O_2_ than high DN electrolytes, so that they can support higher charging currents and are potentially suitable for fast-charging LOBs. However, they also induce more severe side reactions, which passivate the cathode within a few minutes. The composition of these passivation films is mainly the de-composition products of the electrolyte. A high Li_2_O_2_ concentration may be responsible for the high decomposition rate. Thus, the trade-off between the charging reaction rate and electrolyte stability is a key issue to be considered when designing high performance LOB electrolytes. None of the electrolyte investigated in this study exhibits satisfying stability under common LOB working conditions.

For future studies, it is necessary to develop a novel electrolyte that is more stable in the presence of Li_2_O_2_. The RDE method introduced in this study is a facile and informative technique to screen for high performance electrolyte recipes. RDE can not only intuitively detect the oxidation efficiency of dissolved Li_2_O_2_, but also reflect the side reactions. More importantly, this method makes it easier to study the effects of changes in electrolytes, which can be used as a reference for future studies.

## Conflicts of interest

The authors declare that they have no conflict of interest.

## Supplementary Material

RA-008-C8RA03416H-s001
