# Supplementary material for: Rotating-disk electrode analysis of the oxidation behavior of dissolved Li2O2 in Li–O2 batteries
Source: RSC Adv. 2018 Aug 10;8(50):28496–502. doi: 10.1039/c8ra03416h (PMC9083919; doi:10.1039/c8ra03416h)
Supplement: RA-008-C8RA03416H-s001 [file RA-008-C8RA03416H-s001.pdf]

Electronic Supplementary Material (ESI) for RSC Advances.
This journal is © The Royal Society of Chemistry 2018

Supporting Information

Rotating-disk electrode analysis of the oxidation behavior of dissolved Li2O2 in Li-O2 batteries

Jing Ren,[a] Zhimei Huang,[a] Pramod K. Kalambate,[a] Yue Shen[*a] and Yunhui Huang[*a]

a. State Key Laboratory of Material Processing and Die & Mould Technology, School of Materials Science and Engineering, Huazhong University of Science and Technology (HUST), Wuhan 430074, Hubei, P. R. China.
E-mail: shenyue1213@hust.edu.cn (Yue Shen), huangyh@hust.edu.cn (Yunhui Huang)

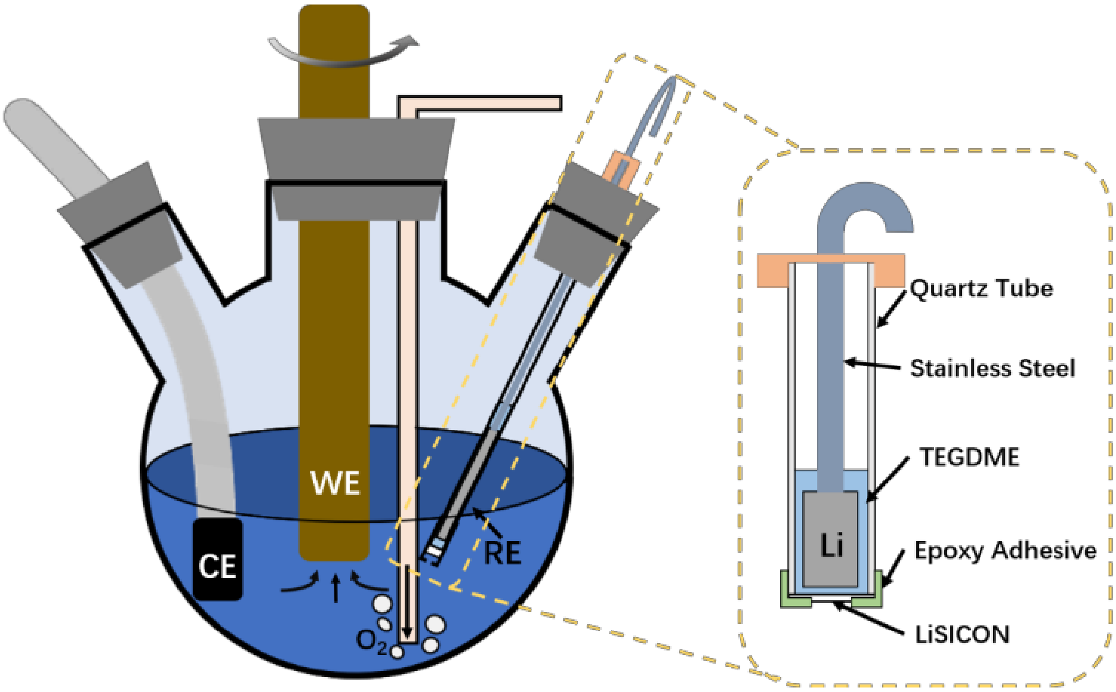

Figure S1 Schematic illustration of three-electrode system and detail of the reference electrode.

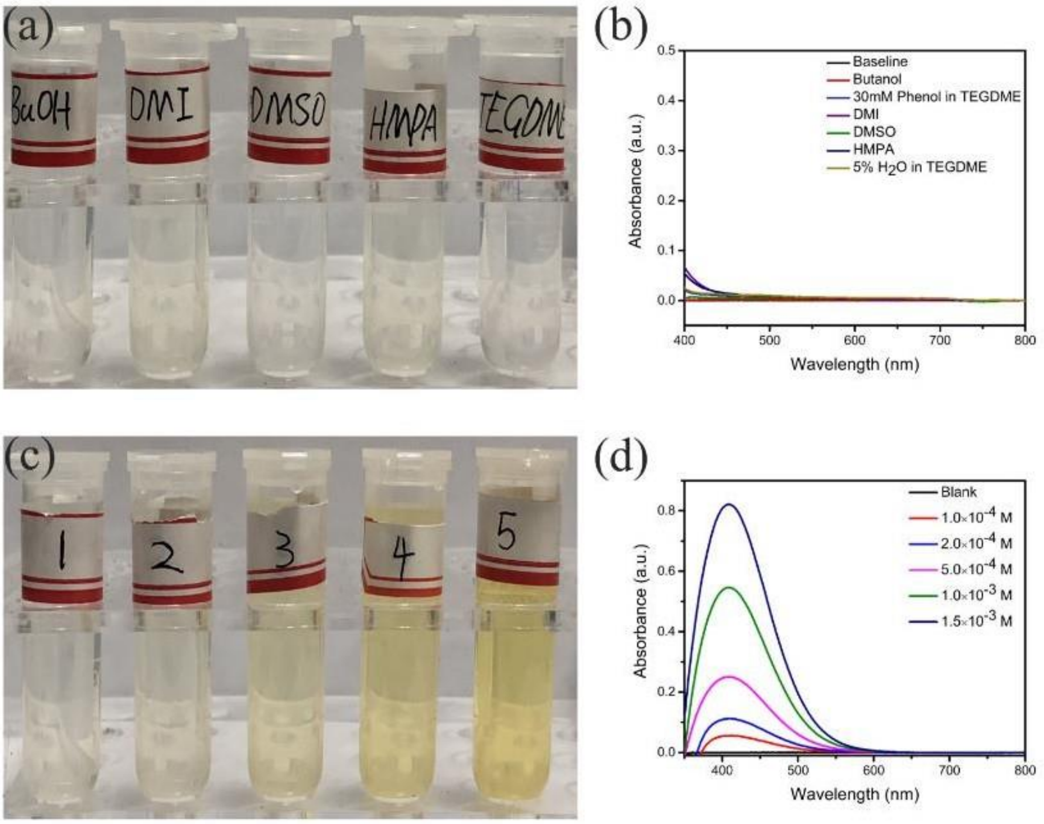

Figure S2 Digital photo (a,c) and UV-Vis absorption spectrum (b,d) of pure electrolytes mixed with $TiOSO_4/H_2SO_4$ solution (a,b) and five standard samples (c,d).

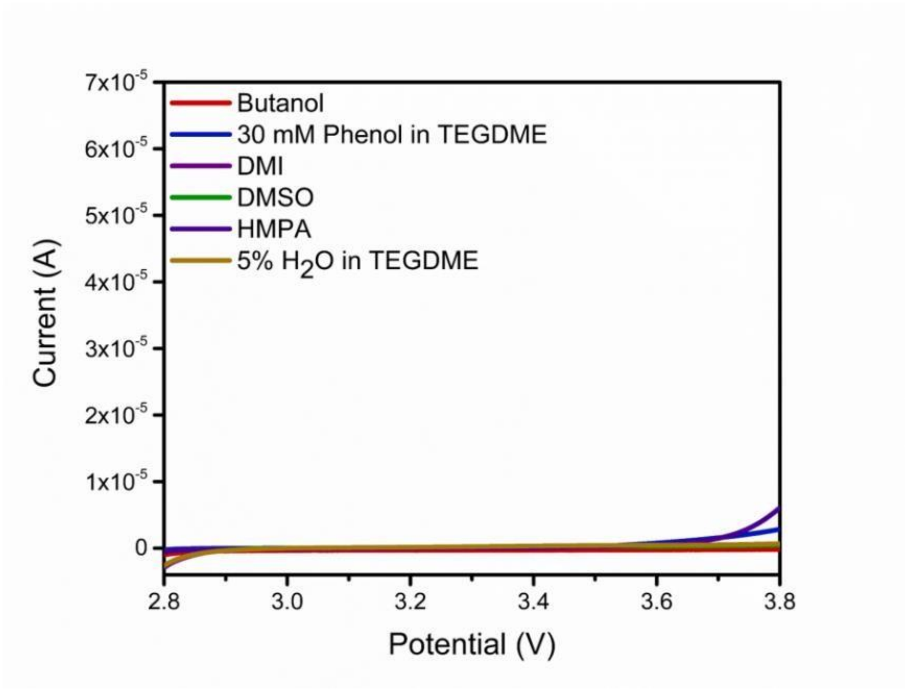

Figure S3 LSV curves of pure electrolytes at the potential range of 2.8 - 3.8 V.

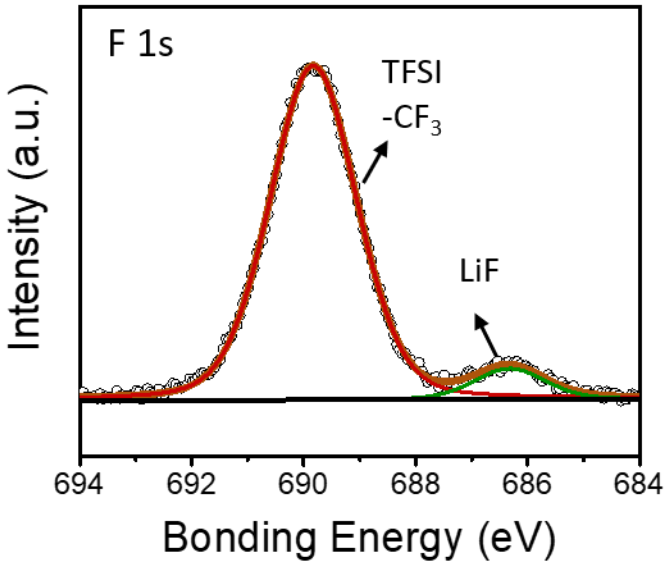

Figure S4 F 1s XPS spectra of the electrode surface in butanol.
